# Reproducibility and Robustness of Graph Measures of the Associative-Semantic Network

**DOI:** 10.1371/journal.pone.0115215

**Published:** 2014-12-12

**Authors:** Yu Wang, Natalie Nelissen, Katarzyna Adamczuk, An-Sofie De Weer, Mathieu Vandenbulcke, Stefan Sunaert, Rik Vandenberghe, Patrick Dupont

**Affiliations:** 1 Laboratory for Cognitive Neurology, Department of Neurosciences, KU Leuven, Leuven, Belgium; 2 Neurology Department, University Hospitals Leuven, Leuven, Belgium; 3 Psychiatry Department, University Hospitals Leuven, Leuven, Belgium; 4 Medical Imaging Research Center (MIRC), University of Leuven and University Hospitals Leuven, Leuven, Belgium; 5 Radiology Department, University Hospitals Leuven, Leuven, Belgium; 6 Alzheimer Research Centre KU Leuven, Leuven Institute for Neuroscience and Disease, Leuven, Belgium; Wake Forest School of Medicine, United States of America

## Abstract

Graph analysis is a promising tool to quantify brain connectivity. However, an essential requirement is that the graph measures are reproducible and robust. We have studied the reproducibility and robustness of various graph measures in group based and in individual binary and weighted networks derived from a task fMRI experiment during explicit associative-semantic processing of words and pictures. The nodes of the network were defined using an independent study and the connectivity was based on the partial correlation of the time series between any pair of nodes. The results showed that in case of binary networks, global graph measures exhibit a good reproducibility and robustness for networks which are not too sparse and these figures of merit depend on the graph measure and on the density of the network. Furthermore, group based binary networks should be derived from groups of sufficient size and the lower the density the more subjects are required to obtain robust values. Local graph measures are very variable in terms of reproducibility and should be interpreted with care. For weighted networks, we found good reproducibility (average test-retest variability <5% and ICC values >0.4) when using subject specific networks and this will allow us to relate network properties to individual subject information.

## Introduction

There is a surge of interest in mapping and modelling the complicated networks within the brain. Functional connectivity analyses of neuroimaging data are based on the concept of synchrony between the signal responses in spatially distinct brain regions [1]. Analysing networks extracted from functional imaging data has proven to be a promising tool to investigate the complex functional structure of the human brain that influences the dynamics underlying cognition [2]–[4].

A promising tool to rigorously study the problem is graph analysis. This provides a framework to characterize and to quantify networks [5]–[9]. Many non-trivial graph characteristics, such as small-worldness, modularity and highly connected hubs, have been observed in human brain networks. Differences in graph properties have been found in people with Alzheimer's disease [10] and schizophrenia [6], [11], [12], and also in association with age [7], [13]. Changes in graph measures were also found during motor learning [14] and taking nicotine [15]. All these studies suggest that graph analysis can be a promising tool in clinical and basic research to characterize brain connectivity in a way that is both biologically meaningful and related to normal and abnormal function. However, an essential requirement when using this type of quantification is that the different measures are reproducible and robust.

The reproducibility of graph measures has already been investigated in a number of studies looking at binarized networks derived from structural MRI [16], [17], diffusion-weighted MRI [18]–[21], resting state fMRI [10], [22], [23], MEG [24] and resting-state functional near infrared spectroscopy [25]. Only one study has looked at the reproducibility of graph measures when using task fMRI [23]. Graph measures using task fMRI is expected to be different compared to resting state fMRI since functional connectivity between two nodes depend on the context (i.e. resting state versus a specific task context). Furthermore, the reproducibility of weighted graph measures has received very little attention: only two studies are available which addressed this problem and both were using graphs derived from diffusion-weighted MRI [26], [27]. Therefore, we investigated the reproducibility and the robustness of graph measures of weighted and binarized networks derived from a task fMRI during explicit associative-semantic processing of words and pictures. This task activates a distributed set of brain areas that has been replicated across a wide range of studies [28]–[38]. Previously, we have applied graph analysis to examine the structure of this network [39].

## Materials and Methods

### Participants

A group of 54 healthy elderly participants (age (mean 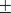 std): 65.2 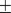 5.6 yrs; 31 male) [40] performed an associative-semantic judgement task. Twenty-eight subjects were scanned on a 3T Philips Intera system equipped with an 8-channel receive-only head coil (Philips SENSitivity Encoding head coil). Twenty-six subjects could not undergo the fMRI in the Intera system due to space limitation in the scanner lumen in combination with the screen. These subjects were scanned on a 3T Philips Achieva system equipped with a 32-channel receive-only head coil (Philips 10 SENSitivity Encoding head coil) which used a screen placed behind the individual's head for the projection.

The protocol was approved by the Ethics Committee University Hospitals Leuven (EudraCT: 2009-014475-45) and written informed consent was obtained from all subjects in accordance with the Declaration of Helsinki.

### Experimental design

Stimuli were projected onto a screen (resolution of 1024×768 pixels, refresh rate 60 Hz) using Presentation 14.8 (NeuroBehavioural Systems, Albany, CA, USA). The design of the fMRI experiment was factorial [28]–[31]. The first factor, task, had two levels: associative-semantic versus visuoperceptual judgement. The second factor, input modality, also had two levels: pictures versus printed words. During a trial of the associative-semantic condition, a triplet of stimuli was presented for 5250 ms, one stimulus on top (the sample stimulus) and one in each lower quadrant (the test stimuli), at 4.6° eccentricity, followed by a 1500 ms interstimulus interval. Subjects had to press a left- or right-hand key depending on which of the two test stimuli matched the sample stimulus more closely in meaning. A given triplet was presented either as pictures or as words and this was counterbalanced across subjects. In the visuoperceptual control condition, a picture or word stimulus was presented in three different sizes (mean picture size was 3.7° and mean letter size 1.2°). Subjects had to press a left- or right-hand key depending on which of the two test stimuli matched the sample stimulus more closely in size on the screen. An epoch, i.e. a block of trials belonging to the same condition, consisted of four trials (total duration 27 s). The fifth condition consisted of a resting baseline condition during which a fixation point was presented in the centre of the screen. During each fMRI run (5 runs in total), a series of the 5 epoch types, was replicated 3 times. The order of conditions was pseudorandom and differed across runs of the same subject. Subjects received a practice session before entering the scanner. In this session we determined which size difference (9%, 6%, 3%, or 1%) for the visuoperceptual conditions was needed for each individual subject to obtain comparable accuracies as for the associative-semantic conditions.

### Preprocessing of the data

Image analysis was performed using Statistical Parametric Mapping (SPM8, Wellcome Department of Cognitive Neurology, London, UK. http://www.fil.ion.ucl.ac.uk/spm). Functional images of each subject were realigned to correct for small head motion during each run. The anatomical 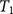-weighted image was coregistered to the average of the realigned functional volumes and non-linearly normalized to Montreal Neurological Institute (MNI) space using the unified segmentation approach [41] and the resulting transformation was used to spatially normalize the functional images. The voxel size of the images in MNI space was 3×3×3 mm^3^. Images were smoothed using a 6×6×6 mm^3^ Full Width at Half Maximum (FWHM) Gaussian kernel. We also applied a temporal high-pass filter (cutoff 270s) and a low-pass filter consisting of the canonical hemodynamic response function. The epoch-related response was modelled by a canonical hemodynamic response function convolved with a boxcar.

### Network construction

Volumes of interests (VOI) were taken from a previously published study on the associative-semantic network [39], namely fifty-seven spheres (radius 6 mm) located at least 20 mm apart. The spheres were centred on group-specific activation maxima (from the main effect of task) determined from this previous study. Note that the position of the VOIs was identical as in the previous study, i.e. the functional information in the current study was not used to position the VOIs. We have previously shown that the nodes of the associative semantic network have a low anatomical inter-subject variability [42].

For each subject, we applied each of these VOIs to the current dataset and we extracted the time series after whitening, filtering and removing effects of no interest (session specific effects) using code from statistical parametric mapping software (SPM8; Wellcome Department of Cognitive Neurology, London, UK; http://www.fil.ion.ucl.ac.uk/spm). Finally, the average time series in the VOI was calculated as the mean of the time series over all voxels in the VOI. Time series of different runs were concatenated. It is important to note that we used the whole time series, i.e. it includes all the different conditions as well as the null condition.

Based on the average time series, partial correlation coefficients between volumes of interest were calculated. Partial correlation was used to obtain the degree of association between regions, with the effect of other regions removed [43], [44]. Among the methods evaluating functional interdependencies between functional MRI time courses in different regions, partial correlations have a high sensitivity to network connection detection [44]. An association matrix was defined in which each element represents the association strength between two regions. The association strength is defined as the absolute value of the z-score which is calculated from the partial correlation using the Fisher r-to-z transform [45]: 
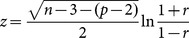
(1)in which 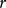 is the partial correlation, 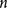 the number of data points in the time series and 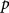 the number of nodes.

The great majority of network analysis in systems neuroscience relies on the construction of undirected and unweighted graphs through thresholding of the association matrix [3], [4]. The thresholded association matrix represents a binary adjacency matrix with 1 indicating the presence and 0 indicating the absence of an edge (connection) between two vertices (nodes/regions). A possible approach to define the threshold is to fix the network's edge density (also referred to as wiring cost), i.e. the number of existing edges divided by the number of possible edges. In order to investigate changes in the network topology as a function of network density, we thresholded the association matrix at network densities ranging from 5% to 45%, in steps of 5%. Densities below 5% are considered too sparse and densities above 50% are less likely to be biological [46], [47]. Furthermore, we also included a density of 7.8% corresponding to the density of the associative-semantic network in the previously published study [39].

The binarization of connections has one major drawback: it enhances scale contrast by underrating (overrating) connections because connections around the threshold may vary considerably between subjects. To avoid this problem, weighted graph analysis [11], [48]–[51], which preserves all the edge information, is also used. To obtain weights 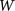 with 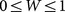, we applied a nonlinear mapping of z score to weight: 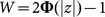
(2)where 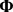 is the cumulative distribution function of the standard normal distribution.

### Graph-theoretical analysis

Local and global graph measures were calculated for the binary network (at different densities) as well as for the weighted network using the brain connectivity toolbox version 2013_12_25 (https://sites.google.com/a/brain-connectivity-toolbox.net/bct/Home; [4]). We calculated the following local graph measures for node 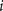: node degree 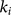, average path length 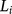, local clustering coefficient 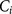, local efficiency 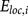, efficiency 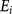 and betweenness centrality 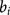. Global measures included characteristic path length 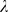, mean clustering coefficient 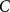, mean local efficiency 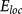, global efficiency 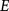 and mean betweenness centrality 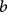. For the definition of these network measures, we refer to [4]. It should be noted that isolated nodes can be present when the density of the network is very low. In that case, these nodes were not taking into account when calculating the network measures.

The network itself was either defined at the individual level (for every subject separately) or at the group level (after averaging the association matrices across the subjects belonging to that group).

### Reproducibility at the individual level

To look at the intra-subject reproducibility, two groups were constructed by evenly splitting each subject's time series into two parts by randomly assigning four of the five runs to one of the two even parts. This corresponds to the situation in which each subject is measured twice under the same conditions. In this way we constructed two groups of paired subjects. We refer to this situation as the *split-half* case. A partial correlation based network and corresponding graph measures were obtained for each subject in each group across a range of densities or using the weights of the network.

The intraclass correlation coefficient (ICC) was used to analyse reproducibility of the network [52]. More specifically, values were merged into a 2×54 matrix (number of measurements x subjects). The total variance was split into the between-subject (BMS) and the residual (EMS) variance. ICC values were calculated according to the equation [53]: 
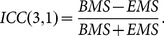
(3)


By randomly switching (100 random realizations) time series parts between the two measurements, we can calculate the mean and variance of the ICC for each graph measure. ICC 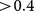 is usually considered as a cut-off for a fairly reliable measure [54].

We also calculated the test-retest value between the two measurements of the same subject and averaged this over all subjects to obtain the average test-retest value. The test-retest 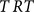 was calculated as: 
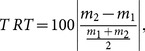
(4)where 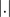 denotes the absolute value and 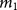 and 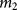 are the values of the graph measure under investigation obtained in the first and second measurement respectively.

### Reproducibility for group-based graph measures

Averaging the association matrices across subjects summarizes the overall characteristics of the group [55], [56]. To study the reproducibility for group-based graph measures, we looked at the test-retest 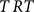 calculated as equation 4 except that 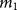 and 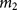 represent the measures obtained for the first and second group. Mean and variance of each graph measure were calculated based upon the 100 random realizations of switching time series parts.

However, a more challenging situation occurs when we want to compare two independent groups which are scanned on MR scanners with different field strength and which differ slightly in the fMRI paradigm. This is the case when we want to compare the results of the current group with the results of our previous study [39]. In that study we used the same paradigm (except that we did not include a null condition in our measurement). Furthermore, the data of this group (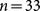) were acquired on a 1.5 T Siemens Sonata. Age (67.2 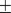 8.5 years) and gender (19 M/14 F) were similar to the current study. We refer in the remaining of the paper to the comparison of these two studies as the *between-independent groups* case. The test-retest values were calculated according to equation 4.

### Hubs and community structure

We also assessed the reproducibility of the community structure and the identification of hubs for binary and weighted networks at the individual and the group level.

The identification of hubs was based on a hub score [2], [11], [14], [39], [57]–[59], which is the sum of dummy values for four criteria. We gave a score of 1 or 0 depending on whether or not the node belongs to the top 20% of nodes with 1) the highest node degree, 2) the highest betweenness centrality, 3) the lowest local cluster coefficient (limited to nodes with a degree 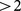), and 4) the lowest average path length. Nodes with a hub score 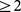 were considered hubs.

We evaluated the consistency of hubs by measuring the co-occurrence of hubs (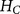) across networks. If 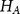 is a list of hubs in network A and 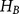 in network B, the co-occurrence is calculated as 
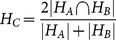
(5)where 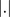 denotes the cardinality of the set. A value of 1 corresponds to a perfect agreement of hubs while 0 reflects no agreement at all.

To determine the community structure of the network, we used the algorithm of Newman [60] as implemented in the Brain Connectivity Toolbox. The algorithm starts from a random order of the nodes which lead to a slightly different community structure each time the algorithm is applied. To generate a consensus assignment of nodes to communities, we used a two step procedure [61]. In the first step, we computed a co-assignment matrix represented as an 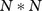 matrix, where cell 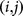 was equal to 1 if node i and node j were assigned to the same community. During the second step, a probabilistic co-assignment matrix is then obtained by averaging 100 realizations of co-assignment matrices.

The consistency of the community structure of the network was assessed by probabilistic scaled inclusivity (pSI), a metric quantifying the consistency of communities across multiple networks and which is an extension of the scaled inclusivity SI [62], [63]. SI is calculated by measuring the overlap of modules across multiple networks while penalizing for disjunction of modules. For example, a node 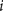 is part of module 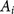 in network A and module 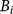 in network B. Then SI for node 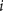, denoted as 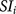, is calculated as 
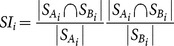
(6)where 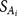 and 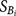 denote sets of nodes in modules 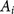 and 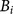 and 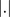 denotes the cardinality of a set. If the two modules 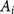 and 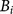 consist of an identical set of nodes, then 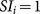. The SI value between two networks is a value between 0 and 1; if 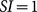 at a particular node, it means that the node is in the same module with exactly the same set of nodes in the two networks. A problem with this way of calculating the SI value is that it requires a final assignment of nodes to a community. This can be done based upon the probabilistic co-assignment matrix but may lead to different results depending on the algorithm to assign the final community to each node. An alternative is to use the probabilistic co-assignment matrix directly to calculate a probabilistic SI value. This is done as follows: 
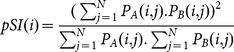
(7)in which 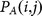 and 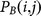 are the probability co-assignment between nodes 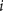 and 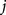 in network 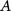 and network 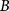.

To further characterize the consistent parts of the communities, we averaged the co-assignment matrices for the two groups in the *between-independent groups* case or across every possible pair in the *split-half* case.

There's no established rule to define which 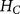 and 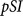 are sufficiently high to ensure consistency between networks but we can determine if it is significantly different from the value obtained under random conditions by comparing it to the distribution of values obtained from null networks, i.e. networks with the same number of nodes and connections which were generated by randomly (1000 realizations) rewiring the observed network [64]. The weighted null network is obtained by randomly rewiring connections with the same distribution of weights.

### Robustness of the data

#### Group size effect

We applied a bootstrapping procedure (100 realizations) to calculate graph measures as a function of group size. More specifically, we created random subsamples from our 54 subjects, each time creating a subgroup with a certain number of subjects, and we repeated this for subgroup sizes ranging from 10 to 53. Results were calculated as the relative change (in %) taking the values of the complete group of 54 subjects as the reference.

#### Network robustness

When we identify the nodes of the network, we may not have captured all nodes. The question then arises, in how far is this affecting the quantification of the network. To address this question, we assume that the 57 nodes represent all nodes of the underlying network and we removed nodes from this network to investigate the impact when nodes were not captured. The procedure is similar to network robustness analysis against random failures and targeted attacks [5], [17], [65] although the interpretation is clearly different. Since it is more likely to miss the least significant nodes, we conducted our analysis by removing nodes based on their significance in the main effect of task in the fMRI study starting by removing the least significant ones. The degree of tolerance will be expressed as the relative change of the graph measures compared to values of the network with all 57 nodes.

### Statistics

To evaluate if ICC values were significantly higher than 0.4, we performed a one-sample t-test. The same test was used to evaluate if the test-retest variability was 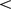 5% or 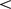 10%. The comparison between subject-specific graph measures obtained in two independent groups was assessed by a two-sample t-test. To test the relation between test-retest variability of global graph measures and density, we first log-transformed the test-retest values and performed a linear regression.

The statistical threshold to reach significance was set to 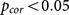. We corrected for the number of densities under investigation in case of global graph measures and additionally for the number of nodes in case of local graph measures.

## Results

In table 1 a summary of the main findings is given.

**Table 1:** Summary of the main findings.

**Subject-specific networks**
Binary networks
• Global efficiency, characteristic path length and mean betweenness centrality are reproducible only when the network density is high.
• The intra subject split-half test-retest values of global graph measures decreases with the increase of density.
Weighted networks
• Global graph measures are reproducible for all the measures investigated.
• The intra subject split-half test-retest values of global graph measures were very low.
• The test-retest values of the mean of the global graph measures derived from subject-specific weighted networks for two independent groups varied between 7 and 17%.
• Communities are consistent for both intra-subject (in the split-half case) and inter-subject comparisons.
• The average global graph measures are not critically depending on the group size.
• The average global graph measures show robustness against missing nodes.
**Group-based networks**
Binary and weighted networks
• In the split-half case, all graph measures show test-retest variability 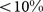
• Hubs show a significant high consistency in the split-half case compared to the values obtained from random null networks.
• Communities show consistency for both the split-half case and when comparing independent groups
• A sufficiently large group size is required to obtain reliable results.
• Global efficiency and characteristic path length are more robust for the group size.
• Global efficiency, characteristic path length, mean local efficiency and clustering coefficient are more robust against missing nodes compared to the mean betweenness centrality
Binary networks
• Test-retest variability of global graph measures decreases as the network becomes more dense.
Weighted networks
• The global efficiency and the characteristic path length have the smallest overall test-retest variability.

### Reproducibility at the individual level (subject-specific networks)

**Figure 1:**
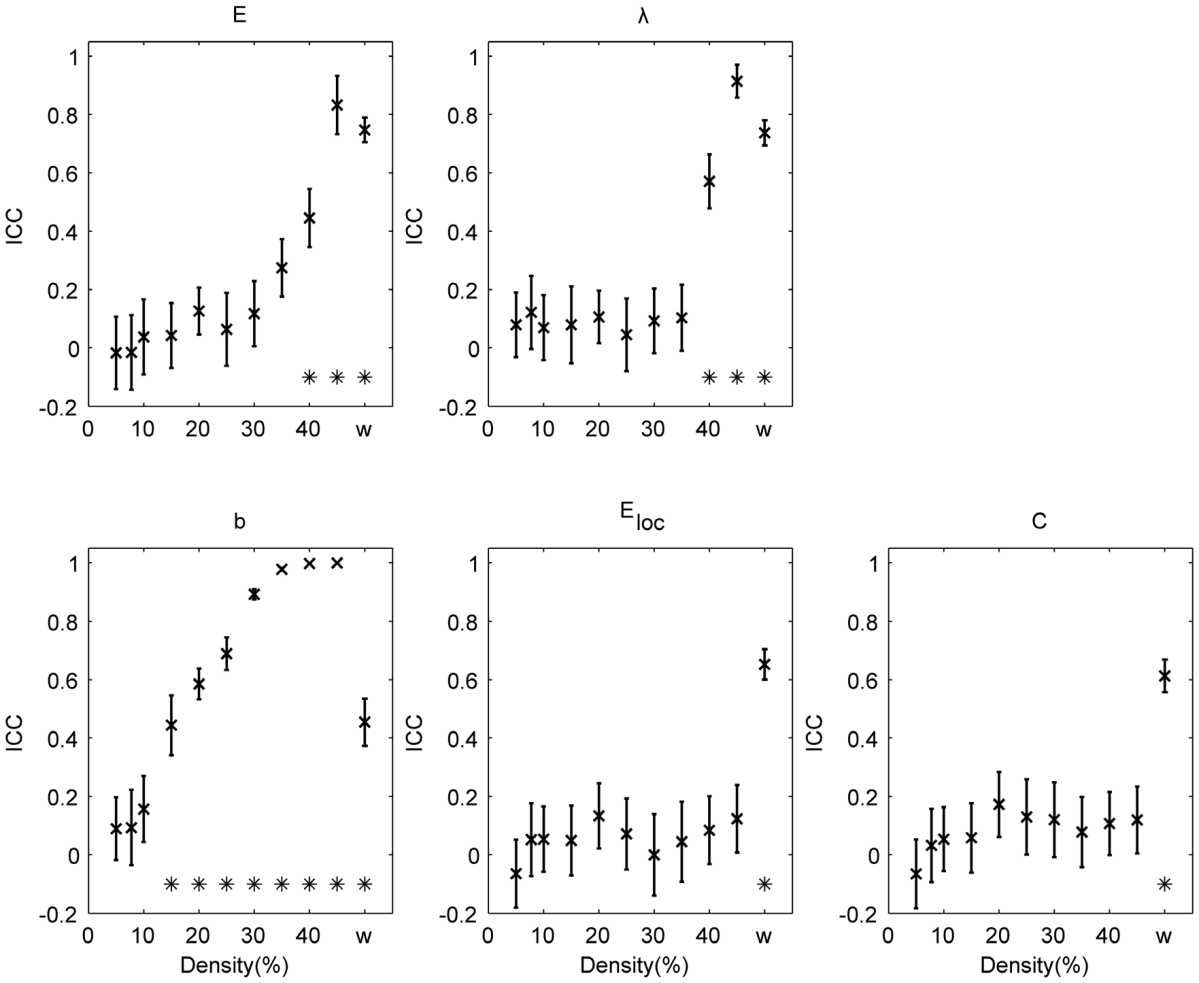
Reproducibility at the individual level. ICC for the global efficiency (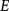), the characteristic path length (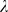), the mean betweenness centrality (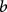), the mean local efficiency (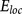) and the mean clustering coefficient (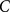). The results are shown for binary (over a range of densities) and weighted (w) networks. Error bars denote the standard deviation. Values significantly (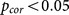) higher than 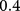 are indicated with *.

Averaged global ICC across all randomizations are shown in Fig. 1 for binary (over a range of densities) and weighted networks. For binary networks, global efficiency 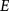, characteristic path length 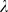 and mean betweenness centrality 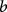 show significant (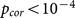) reproducibility (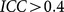) when the network density is high (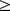
40%). This is not the case for the mean cluster coefficient and the mean local efficiency. Weighted global graph measures show significant (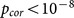) reproducibility (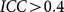) for all the measures investigated (Fig. 1).

As can be expected, reproducibility at the nodal level exhibits heterogeneity across graph measures and nodes (see S1 Table). Efficiency 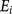 and average path length 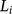 are the most reproducible nodal graph measures (although only in 2 nodes a significant (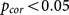) ICC value 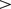 0.4 was found) in contrast to the betweenness centrality 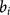 which is the least reproducible one. In case of weighted local graph measures, we observed the folowing range of averaged (across subjects) ICC values: 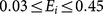; 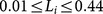; 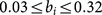; 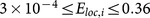 and 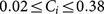.

**Figure 2:**
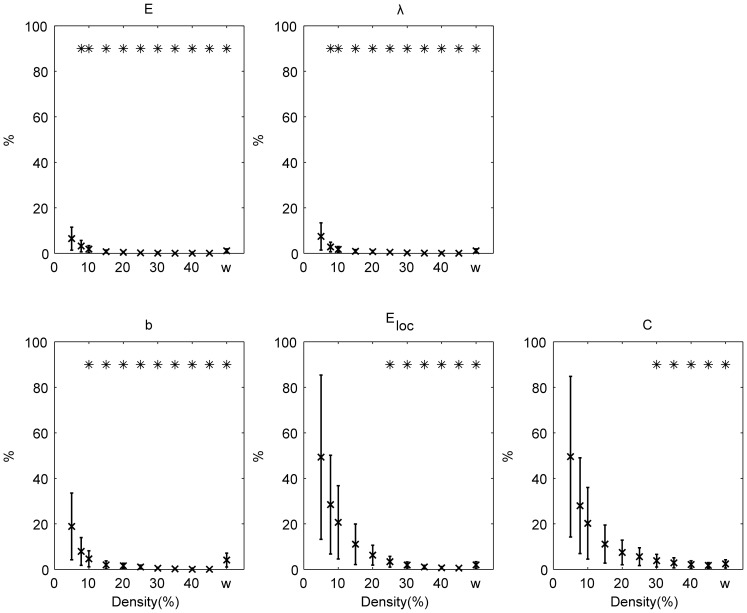
Intra-subject split-half test-retest variability (%). The results are shown for binary (over a range of densities) and weighted (w) networks. Error bars refer to the standard deviation across all randomization and subjects. 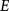: global efficiency; 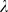: the characteristic path length; 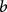: the mean betweenness centrality; 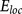: the mean local efficiency; 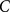: the mean clustering coefficient. Test-retest variabilities significantly (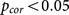) lower than 10% are indicated with *.

The intra subject split-half test-retest values (TRT) for global graph measures are shown for binary and weighted networks (Fig. 2). The test-retest values of global graph measures decreases with the increase of density in case of binary networks (for all global graph measures under investigation 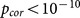). For weighted networks, the test-retest values were excellent (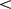 5%): 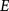:1.12% (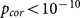);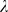: 1.11% (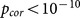); 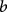: 4.09% (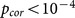); 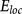: 1.93% (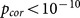) and 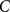: 2.40% (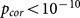).

In table 2, we show the comparison of the global graph measures derived from subject-specific weighted networks for the two independent groups. The values in the current group are significantly different from those obtained in our previous study [39] and the test-retest values of the mean of the global graph measures derived from subject-specific weighted networks for two independent groups varied between 7 and 17% (table 2).

**Table 2:** Comparison of global graph measures derived from subject-specific weighted networks between two independent groups.

	Current study mean 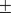 std	Previous study [39] mean 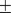 std	TRT of the mean %
mean clustering coefficient	0.588 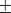 0.020	0.520 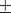 0.022	12
characteristic path length	1.444 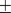 0.028	1.544 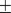 0.034	7
global efficiency	0.739 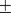 0.014	0.690 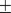 0.016	7
mean local efficiency	0.629 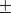 0.018	0.566 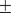 0.020	11
mean betweenness centrality	0.0057 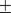 0.0003	0.0067 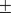 0.0004	17

TRT: test-retest variability.

### Reproducibility for group-based graph measures

**Figure 3:**
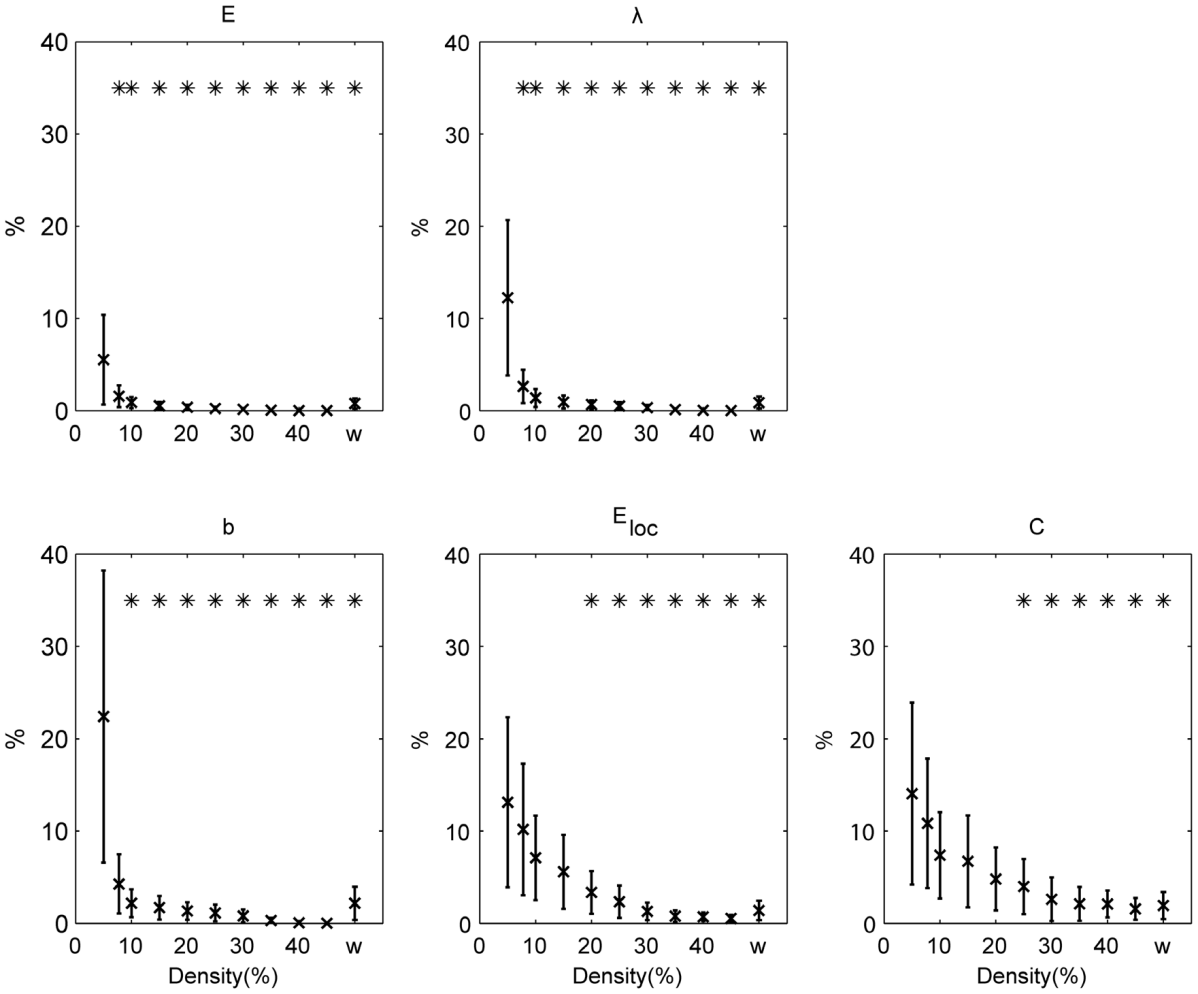
Test-retest variability (%) for the split-half case. The results are shown for binary (over a range of densities) and weighted (w) networks. Error bars refer to the standard deviation. 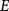: global efficiency; 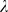: the characteristic path length; 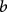: the mean betweenness centrality; 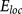: the mean local efficiency; 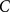: the mean clustering coefficient. Test-retest variabilities significantly (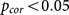) lower than 10% are indicated with *.

**Figure 4:**
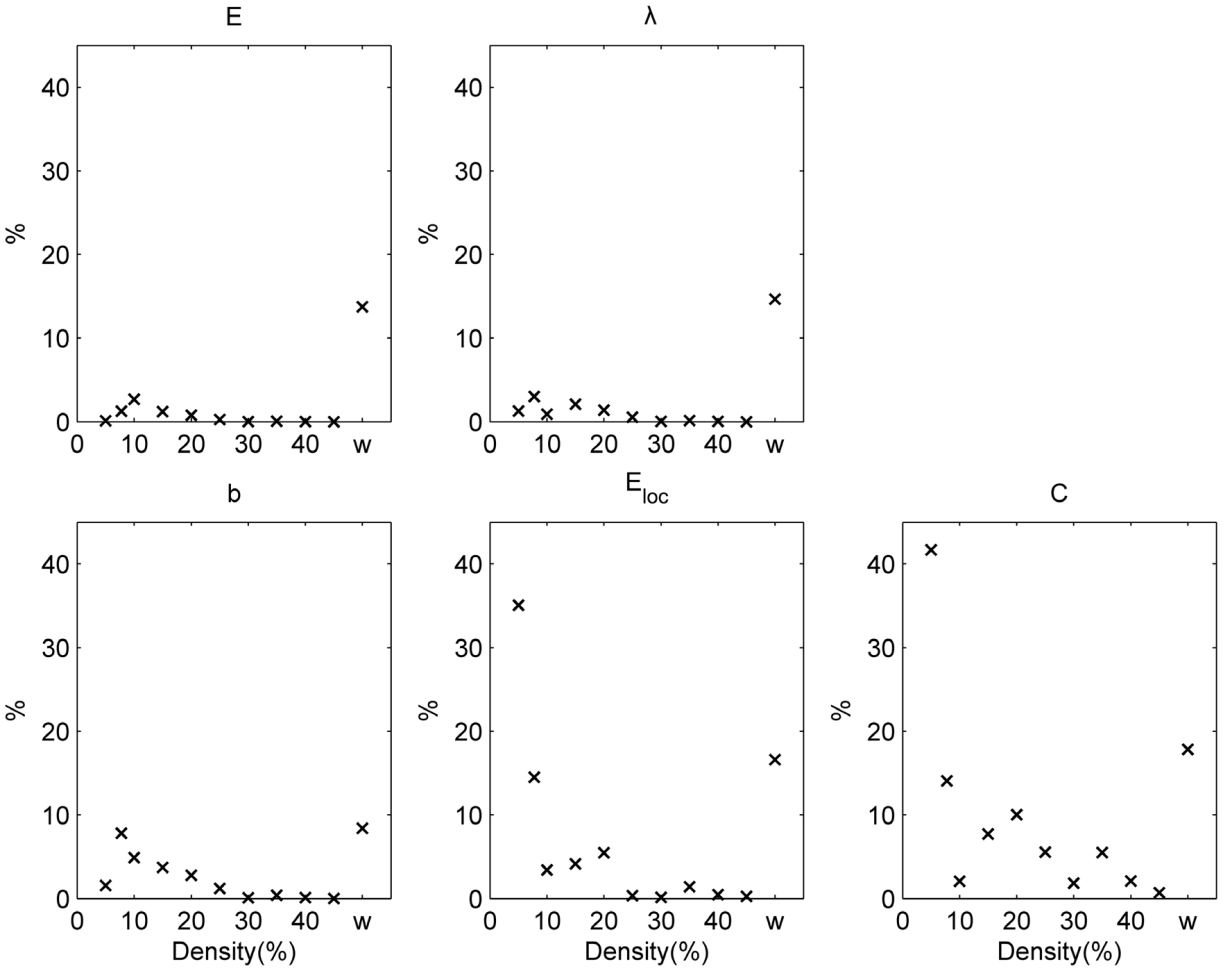
Test-retest variability (%) between independent groups for binary (over a range of densities) and weighted (w) networks. 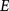
: global efficiency; 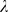: the characteristic path length; 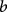: the mean betweenness centrality; 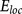: the mean local efficiency; 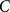: the mean clustering coefficient.

Test-retest variability for the different global graph measures are shown for the *split-half* case (Fig. 3) and the *between-independent groups* case (Fig. 4) for binary and weighted networks.

In the *split-half* case (Fig. 3), all graph measures show a significantly (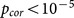) small test-retest variability (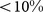) for binary (with densities 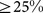) and weighted networks. The global graph measures showing the smallest overall test-retest variability are the global efficiency and the characteristic path length. For binary networks, test-retest variability of global graph measures decreases as the network becomes more dense (for all global graph measures under investigation: 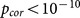). When looking at the local graph measures (table 3), we observe that the efficiency and average path length in the majority of nodes show a significantly (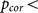 0.05) small test-retest variability (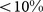) for both binary (with densities 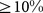) and weighted networks. On the other hand, betweenness centrality has the largest test-retest variability.

**Table 3:** The split-half case: the test-retest variability of local graph measures across densities.

**% of nodes with a significant (** 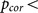 ** 0.05) test-retest variability ** 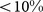											
density%	5	7.8	10	15	20	25	30	35	40	45	w
node degree	28	9	7	0	0	0	0	2	0	0	98
cluster coefficient	9	0	0	0	0	0	4	12	37	75	98
average path length	0	53	91	95	98	100	100	100	100	100	100
efficiency	23	65	89	95	96	100	100	100	100	100	100
local efficiency	7	2	2	2	21	46	79	95	100	100	98
betweenness centrality	0	0	0	0	0	0	0	0	0	0	0
**median test-retest variability (%)**											
density%	5	7.8	10	15	20	25	30	35	40	45	w
node degree	15	18	18	16	16	15	14	13	12	11	5
cluster coefficient	56	41	31	24	20	16	13	10	9	7	6
average path length	15	8	6	5	4	4	3	3	3	3	3
efficiency	11	7	6	5	4	4	3	3	3	3	3
local efficiency	58	46	34	23	14	8	5	4	3	2	6
betweenness centrality	90	69	58	52	49	44	40	37	33	29	42

In the *between-independent groups* situation, there was a trend that the test-retest variability of global graph measures in case of binary networks (Fig. 4) decreases as the network becomes more dense (for all global graph measures under investigation: uncorrected 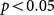). Furthermore, the test-retest variability for graph measures of weighted networks in this situation are mostly above 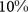. When looking at the local graph measures (table 4), we observe that the efficiency and average path length in the majority of nodes show small test-retest variability (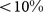) for binary networks with densities 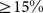.

**Table 4:** Between independent groups: the test-retest variability of local graph measures across densities.

**% of nodes with a test-retest variability ** 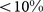											
density%	5	7.8	10	15	20	25	30	35	40	45	w
node degree	26	18	25	19	16	16	23	21	26	35	25
cluster coefficient	7	7	12	14	19	19	32	53	53	75	26
average path length	23	47	46	60	65	72	79	84	81	75	30
efficiency	30	44	53	60	65	68	72	77	72	75	35
local efficiency	4	7	16	26	40	53	70	88	98	100	30
betweenness centrality	7	5	2	4	7	7	11	12	16	16	14
**median test-retest variability (%)**											
density%	5	7.8	10	15	20	25	30	35	40	45	w
node degree	40	40	29	25	27	29	24	20	18	16	18
cluster coefficient	129	73	57	26	26	20	13	9	9	6	19
average path length	18	11	11	8	7	7	5	4	4	5	16
efficiency	17	11	9	8	8	6	6	5	5	5	15
local efficiency	133	79	53	22	17	8	5	3	3	2	18
betweenness centrality	116	118	105	91	60	62	58	52	44	37	67

### Hubs and communities

We analysed the hubs and community structure for binary and weighted networks at the individual and group-averaged level.

At the group-averaged level, table 5 shows the co-occurrence of hubs 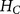 in the *split-half* case and in the comparison between independent groups. For binary and weighted networks, hubs show a significant (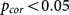) high consistency in the *split-half* case compared to the values obtained from random null networks. However, when comparing two independent groups the consistency of the hubs is clearly reduced.

**Table 5:** Co-occurrence of hubs 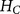.

**Split-half case**											
density%	5	7.8	10	15	20	25	30	35	40	45	w
*H_c_*	**0.62**	**0.70**	**0.78**	**0.75**	**0.73**	**0.70**	**0.68**	**0.66**	**0.64**	**0.62**	**0.79**
**Between-independent groups**											
density%	5	7.8	10	15	20	25	30	35	40	45	w
*H_c_*	**0.43**	**0.32**	**0.52**	**0.31**	**0.31**	**0.30**	**0.41**	**0.30**	**0.34**	**0.40**	**0.43**

*Bold: Values which are significantly (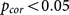) different from the value obtained from null networks (see text). Italic: 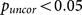.*

The results for the consistency of the community structure are given in table 6 as the mean pSI for both binary and weighted networks. Communities show consistency for both the *split-half* case and when comparing independent groups, i.e. the pSI values are significantly different from those obtained from random null networks. When we look at groups of nodes which are consistently assigned (average value in the co-assignment matrix 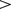 0.95) to the same module between the two independent groups at the density 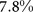, we find the following groups: 1) nodes in the dorsomedial prefrontal gyrus, the left superior frontal gyrus, the left supplementary motor area, the left anterior and the right cingulate gyrus; 2) nodes in the left inferior frontal gyrus pars orbitalis, the left posterior middle temporal gyrus, the left anterior and posterior superior temporal sulcus and the left supramarginal gyrus; 3) nodes in the left lingual gyrus, the left intraparietal sulcus and the left middle occipital gyrus; 4) nodes in the left superior frontal gyrus and the left medial frontal; 5) nodes in the right caudate, the left anterior thalamus and posterior thalamus; 6) nodes in the right inferior and middle occipital gyrus.

**Table 6:** Mean pSI.

**Split-half case**											
density%	5	7.8	10	15	20	25	30	35	40	45	w
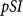	**0.31**	**0.35**	**0.40**	**0.42**	**0.42**	**0.47**	**0.50**	**0.56**	**0.54**	**0.49**	**0.50**
**Between-independent groups**											
density%	5	7.8	10	15	20	25	30	35	40	45	w
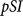	**0.14**	**0.13**	**0.15**	**0.18**	*0.14*	**0.29**	**0.23**	**0.35**	**0.33**	**0.37**	**0.44**

*Bold: Values which are significantly (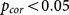) different from the value obtained from null networks (see text). Italic: 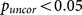.*

At the individual level for weigthed networks in the split-half case, the mean intra-subject co-occurrence of hubs 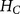 across all 54 subjects and 100 randomization is 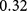 (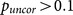) while the mean inter-subject 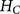 across all pairs of subjects is 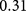 (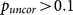). The mean intra-subject consistency of the community structure 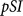 across all 54 subjects and 100 randomization is 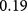 (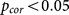) while the mean inter-subject 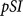 across all pairs of subjects is 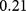 (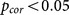).

At the individual level for weigthed networks in the comparison of two independent groups, the mean inter-subject 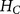 across all pairs of subjects was 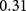 for the current study and this was not significantly different form 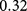 obtained from the data of the previous study [39]. However, the mean inter-subject 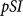 across all pairs of subjects was significantly (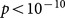) different: 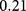 for the current study versus 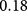 for the previous study [39].

### Group size effect

**Figure 5:**
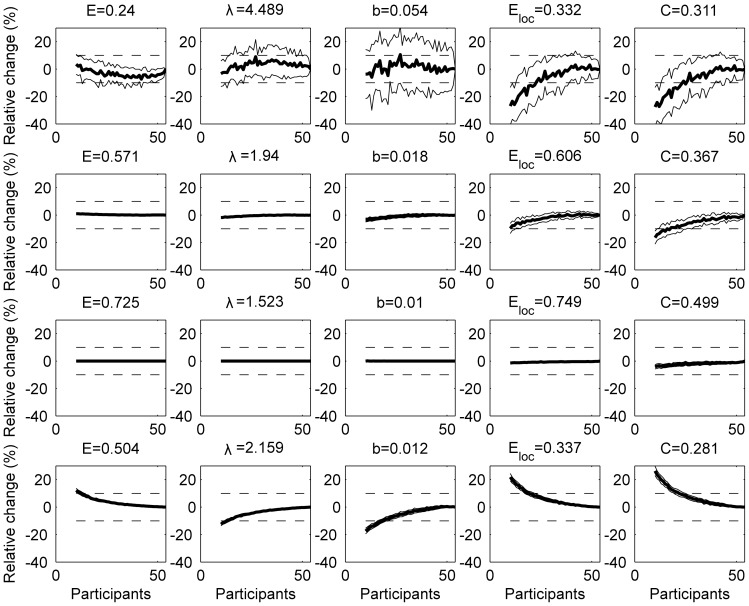
Group size effect for group based networks. The effect of group size for networks at a density of 5% (top row), 20% (second row), 45% (third row) and the weighted network (bottom row). A bootstrapping procedure was used (100 realizations) to randomly group the subjects with increasing group size. For graph measures the relative change (%) to the reference value (which is obtained when taking the complete group) are shown. Full lines denote the mean (bold) and 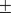 standard deviation of the metric. Dotted lines represent a relative change of 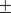 10%. 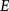: global efficiency; 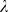: the characteristic path length; 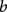: the mean betweenness centrality; 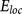: the mean local efficiency and 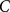: the mean clustering coefficient.

For group based networks, the relative change of global graph measures as a function of group size are shown in Fig. 5 for networks with a density of 5%, 20%, 45% as well as for the weighted network. Overall, the relative difference in graph measures when taken the complete group (54 subjects) as the reference, decreases when the number of participants and/or the density increase. Furthermore, some binary graph measures are more robust for the group size: the absolute value of the relative error of global efficiency and characteristic path length are significantly (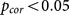) smaller than 10% even for smaller group sizes (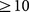 subjects) for all densities 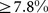. The mean betweenness centrality, the mean local efficiency and the mean clustering coefficient show a similar behaviour but for minimum group sizes of respectively 17, 38 and 44 subjects for a binary network at a density of 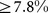. At a density of 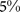, the minimum group size to obtain relative errors significantly (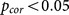) smaller than 10% in absolute value, could only be determined for 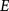, 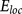, 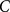 and 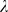 and this size was respectively 42, 44, 44 and 49 subjects. For the weighted network, the minimum group size is 13, 14, 19, 20 and 23 for 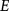, 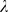, 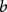, 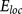 and 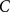 respectively.

**Figure 6:**
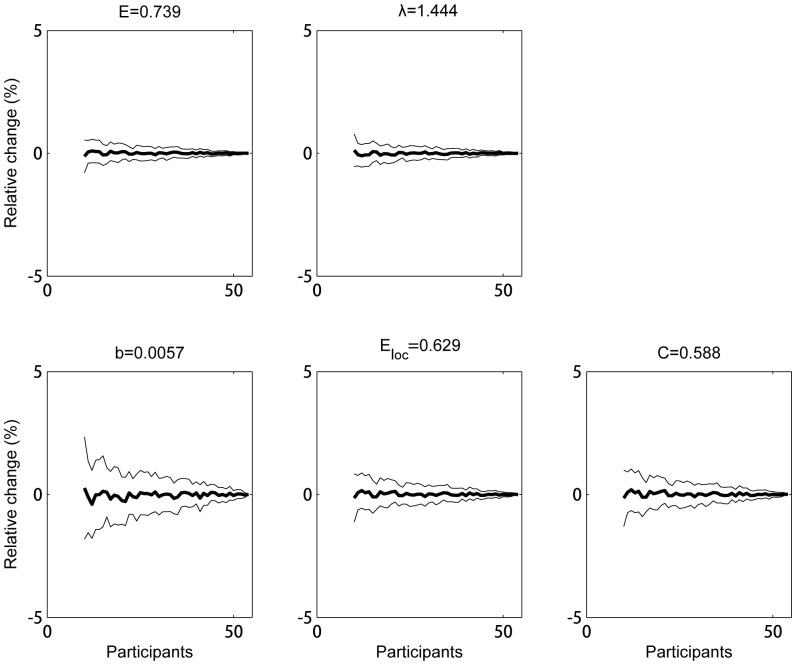
Group size effect for groups of individual networks. The average across subjects of the weighted graph measures determined from the individual's network is shown as function of group size. A bootstrapping procedure was used (100 realizations) to randomly group the subjects with increasing group size. For graph measures the relative change (%) to the reference value (which is obtained by averaging across all subjects) are shown. Full lines denote the mean (bold) 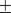 standard deviation of the metric. Dotted lines represent a relative change of 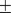 10%. 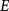: global efficiency; 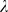: the characteristic path length; 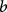: the mean betweenness centrality; 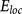: the mean local efficiency and 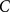: the mean clustering coefficient.

At the individual level for weigthed networks, all the average global graph measures showed a small relative error (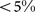 in absolute value, 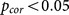) as a result of the smaller group size even for groups as small as 10 subjects (Fig. 6).

### Network robustness

**Figure 7:**
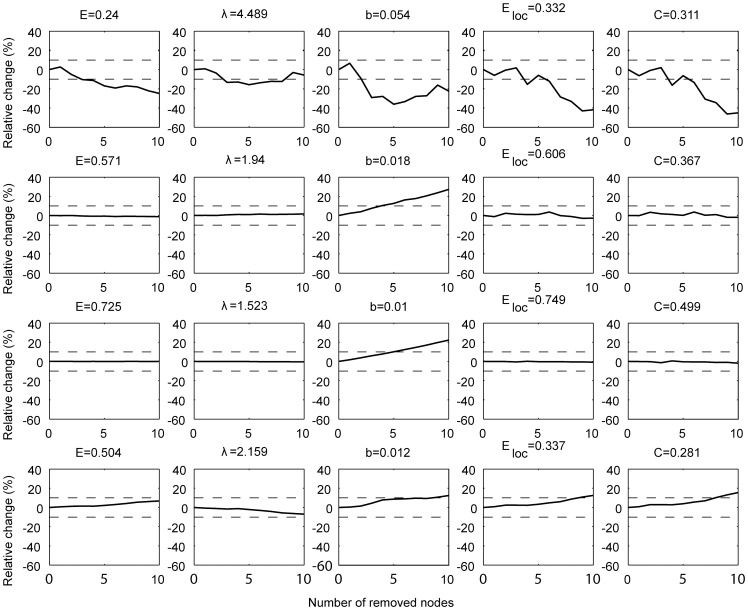
Robustness to missing nodes for networks with an initial density of 5% (top row), 20% (second row), 45% (third row) and the weighted network (bottom row). The relative change (%) to the value obtained when taking the intact network as the reference is shown. The nodes were removed based on their significance in the main effect of task (starting with the least significant ones). Dotted lines indicate the 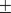 10% interval. Relative changes significantly (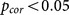) lower than 10% in absolute value are indicated with *.

For group based networks, the robustness of graph measures in case we missed the least significant nodes is shown in Fig. 7 for binary (with an initial density of 5%, 20%, 45%) and weighted networks. The robustness depends on the network measure under investigation. Global efficiency, characteristic path length, mean local efficiency and clustering coefficient are more robust compared to the mean betweenness centrality.

**Figure 8:**
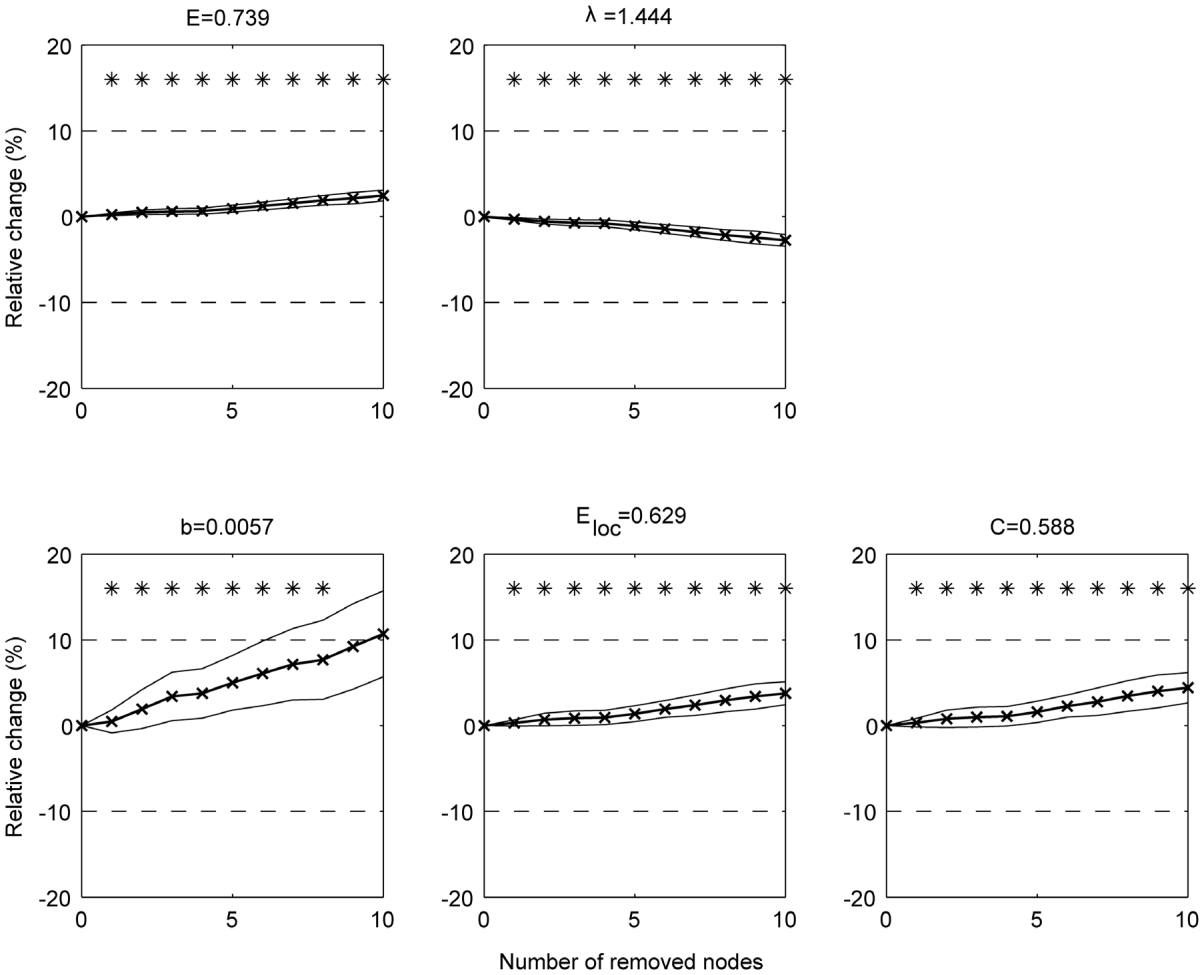
Robustness to missing nodes for individual weighted networks. The relative change (%) to the value obtained when taking the intact network as the reference is shown. The nodes were removed based on their significance in the main effect of task (starting with the least significant ones). Full lines denote the mean (bold) 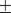 standard deviation of the metric across all subjects.

At the individual level for weigthed networks, the robustness to missing nodes remains within 10% error (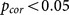) up to removal of the 8 least significant nodes (Fig. 8).

## Discussion

In this work, reproducibility and robustness of the functional connectivity network associated with an associative-semantic task was examined by studying local and global graph measures, hubs and the community structure. The nodes of the associative-semantic network were taken from a previous study [39]. The paradigm that we used gives a highly consistent activation pattern using univariate analyses and this is replicated in our and other centers [28]–[38]. Furthermore, we have shown previously that the nodes have a low anatomical inter-subject variability [42]. This consistency and reproducibility at the nodal level is essential when looking at the network measures.

### Choice of connectivity measure

A network is dependent on the choice of the measure of connectivity between different brain regions. Many groups investigating functional networks in the human brain use the Pearson correlation coefficient [8], [50], [66]. Other similarity measures have also been used, including the correlation between wavelet components [5], [10], [67] and mutual information [44]. However, constructing a network by correlation or mutual information does not necessarily imply that the functional connection between two nodes is direct. The distinction between direct and indirect functional relationships between areas is very important in terms of correctly estimating the network. Hence, partial correlation became a hot topic in recent years (e.g.[43], [68], [69]). Partial correlation provides a convenient summary of conditional independence and turns out to be an effective way to model the connectivity [44]. In our work, partial correlations are used to remove mutual dependencies on common influences from other brain areas. By conditioning the dependencies between two nodes on other nodes, the functional connectivity (i.e. partial correlation) reflects a quantity that is more closely related to direct interaction, taking the analysis of functional connectivity closer to the characterization of functional interactions in terms of effective connectivity. It is data-driven in the sense that, unlike existing methods such as structural equation modelling (SEM) and dynamic causal modelling (DCM), it does not require any prior information regarding functional interactions.

### Split-half variability versus comparison of two independent groups

In this work, we have studied two situations which reflect two complete different situations when looking at the variability of the measurement. The first situation is the one in which we have split the timeseries in two even parts to assess the split-half variability. This corresponds to a situation in which subjects are measured twice under almost similar conditions (i.e. exactly the same scanner, the same sequence, the same paradigm) within one session and assuming no time effects. The limitation of this approach is that we have violated the temporal order of the runs and that the number of runs in the newly composed parts is small. The other situation corresponds to the measurement of two independent groups on different scanners using a slightly different paradigm. As can be expected, the test-retest variability in the latter case is larger than the split-half variability in case of weighted networks and for most densities. The test-retest values of the mean of the global graph measures derived from subject-specific weighted networks for two independent groups varied between 7 and 17%. However, the values were significantly different between both groups most likely due to the inclusion of a null condition in the current study.

### Reproducibility

For binary group based networks, we observed that the reproducibility improves when the density of the network increases. Networks with high density, weighted group based networks and weigthed individual networks show all a very good reprodubility for the global graph measures. Only when we compared two independent groups, the weighted group based network showed a weaker reproducibility. Networks with low density (e.g. 5%), showed weak reproducibility and this was depending on the graph measure itself.

Local graph measures showed weak reproducibility in almost all situations for most nodes and therefore quantification of local graph properties needs to be interpreted with care.

### Hubs and community structure

A node playing a pivotal role in the flow of information is called a hub but the operational definition of a hub differs between studies. In the current study, we have taken a similar approach as [11]. We observe a high co-occurrence in the *split-half* case but a low co-occurrence for the comparison between independent groups. We also observe that the co-occurrence is relatively stable over the different densities and this is also the case for the weighted graph measures. The average inter-subject and intra-subject co-occurrence of hubs in case of individual weighted networks is somewhat lower and this is probably due to the higher variability which one can expect in individual networks compared to group based networks.

The community structure represents how nodes are separated into interacting (integrated) but distinct (segregated) functional modules. A major challenge in examining network module organization is the reproducibility of modules and how to measure this reproducibility. Several studies compared modularity Q and number of communities to achieve this goal [49], [50], [70]. However, the value of Q only gives a sense of the network strength in dividing itself into modules. One could easily have two networks which may be considerably different, yet sharing the same number of communities and similar Q values. A more appropriate measure is to use scaled inclusivity which is a measure for the overlap of modules across networks while penalizing for disjunction of modules [62], [63]. The calculation requires a final assignment of nodes to a community. In this work, we have extended this formula so that it is now directly based on the probabilistic co-assignment matrix without the need to assign each node to a community. In case the co-assignment matrix is binarized (which is similar to assigning each node to a community) it reduces to the original formula. We found that the reproducibility of the community structure of weighted group based networks was similar or even better compared to the values for dense binarized networks. When looking at the average intra- and intersubject probabilistic scaled inclusivity, we observed smaller values most likely again due to the higher variability which you can expect in individual networks compared to group based networks.

### Group size effect

An important issue relates to the number of subjects required to obtain robust graph measures. In a recent study [71], it was emphasized that a small sample size undermines the reproducibility of neuroscience. We found that in low density networks, a large number of subjects is required to obtain robust values and this depends on the graph measure under investigation (global efficiency and characteristic path being the most stable measures). The use of weighted graph measures leads to robust values. This is also true for the averaged graph measures in case of individual weighted networks.

### Robustness against missing nodes

If we have not captured all nodes of the network, the question is in how far graph measures will be influenced. Indeed, some nodes are only weakly activitated in an fMRI experiment and the choice of selection of inclusion of nodes can be based on a statistical criterion. We found that all graph measures, except the mean betweenness centrality, are robust even if we didn't include several of these weaker nodes.

### Binary versus weighted networks

The popularity of binary network analysis may arise from the fact that in most cases it is simpler to characterize[4]. In our previous study [39], we have binarized the network based on a statistical criterion (significant association values). If we would have applied the same criterion in our current study, we would have found much more significant connections (and therefore a higher density) because of the larger cohort size, the inclusion of a null condition and the use of an MRI scanner with a higher magnetic field. Networks with different densities can have different properties [51]. To overcome this problem, we used an equi-density thresholding [67], [72] and we have studied the networks at different densities like most other studies [13], [50], [66], [67]. Another problem with the use of a threshold to binarize the network is that a small change in the association (connectivity) strength can lead to a change in connectivity (just below versus just above a threshold).

To overcome these problems, one can define a weighted network. The question is then how to define these weights. Some groups take the connection with the highest connectivity value and set this to one and scale the remaining connections accordingly [11]. This approach is straightforward and simple but it is also more sensitive to noise. We propose an approach which is based on the fact that the partial correlations were transformed to a Z-score using a Fisher r-to-Z transform and by applying a non-linear transform based on the cumulative distribution of the standard normal distribution. This approach is less sensitive to noise (or to outliers) in connection strength and it leads in a natural way to positive weights between 0 and 1.

### Subject specific versus group based networks

When studying brain networks in a group of subjects we have two possible approaches: 1) define the network for each subject, calculate the variables of interest (e.g. network measures) and use these values for further analysis or 2) define the network based upon the group itself by e.g. averaging the association matrices across the subjects of the group and by calculating the network (and the corresponding measures) based upon this averaged association matrix. The first approach gives information about the individual variability and we can relate directly graph measures to other subject specific information. This is important especially in the light of comparing and quantifying networks in normals and patients. However, the disadvantage is that the results are more sensitive to noise in the measurements. On the other hand, the notion that averaging connection strengths across subjects summarizes the overall characteristics of the group is widely accepted [55], [56].

Based on the ICC values and on the intra-subject test-retest results, weighted individual networks can be realiably determined. The fact that the identification of hubs and communities is more variable compared to the group based networks is less important since it may also be a result of subject specific behaviour and in that sense it may capture the underlying biological variability.

## Conclusions

We have studied the reproducibility and robustness of various graph measures in group based and in individual binary and weighted networks derived from an fMRI experiment using an associative-semantic paradigm. We have shown that global graph measures exhibit a good reproducibility and robustness but the results depend on the graph measure itself and on the density in case of binary networks. Group based binary networks should be derived from groups of sufficient size and the lower the density the more subjects are required to obtain robust values. Local graph measures are very variable in terms of reproducibility and should be interpreted with care. For weighted networks, we found good reproducibility when using subject specific networks and this will allow us to relate network properties to individual subject information.

## Supporting Information

S1 FigureGraph of the associative-semantic network. The connection strength is determined by the partial correlation. Only connections which are significant at uncorrected p
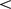
0.05 are shown. The density is 42.6%.(JPG)

S2 FigureGraph of the associative-semantic network. The connection strength is determined by the partial correlation. Only connections which are significant at corrected (for the number of possible connections) p
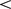
0.05 are shown. The density is 14%.(JPG)

S3 FigureCorrelation between the average contrast values (based on the beta values and the main contrast of task) of any pair of nodes and the strength of the functional connectivity (expressed as the Z-values obtained from the partial correlations after a Fisher r-to-z transform) between these nodes to investigate if there is a relation between GLM results and the likelihood of having an edge. Values are plotted for every connection and every subject. The correlation is weak (r = 0.027) but very significant (p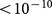).(JPG)

S1 Table ICC values for each node for different graph measures at different densities and for the weighted network.
(XLSX)
