# Supplementary figures and images for: Reproducibility and Robustness of Graph Measures of the Associative-Semantic Network
Source: PLoS One. 2014 Dec 12;9(12):e115215. doi: 10.1371/journal.pone.0115215 (PMC4264875; doi:10.1371/journal.pone.0115215)

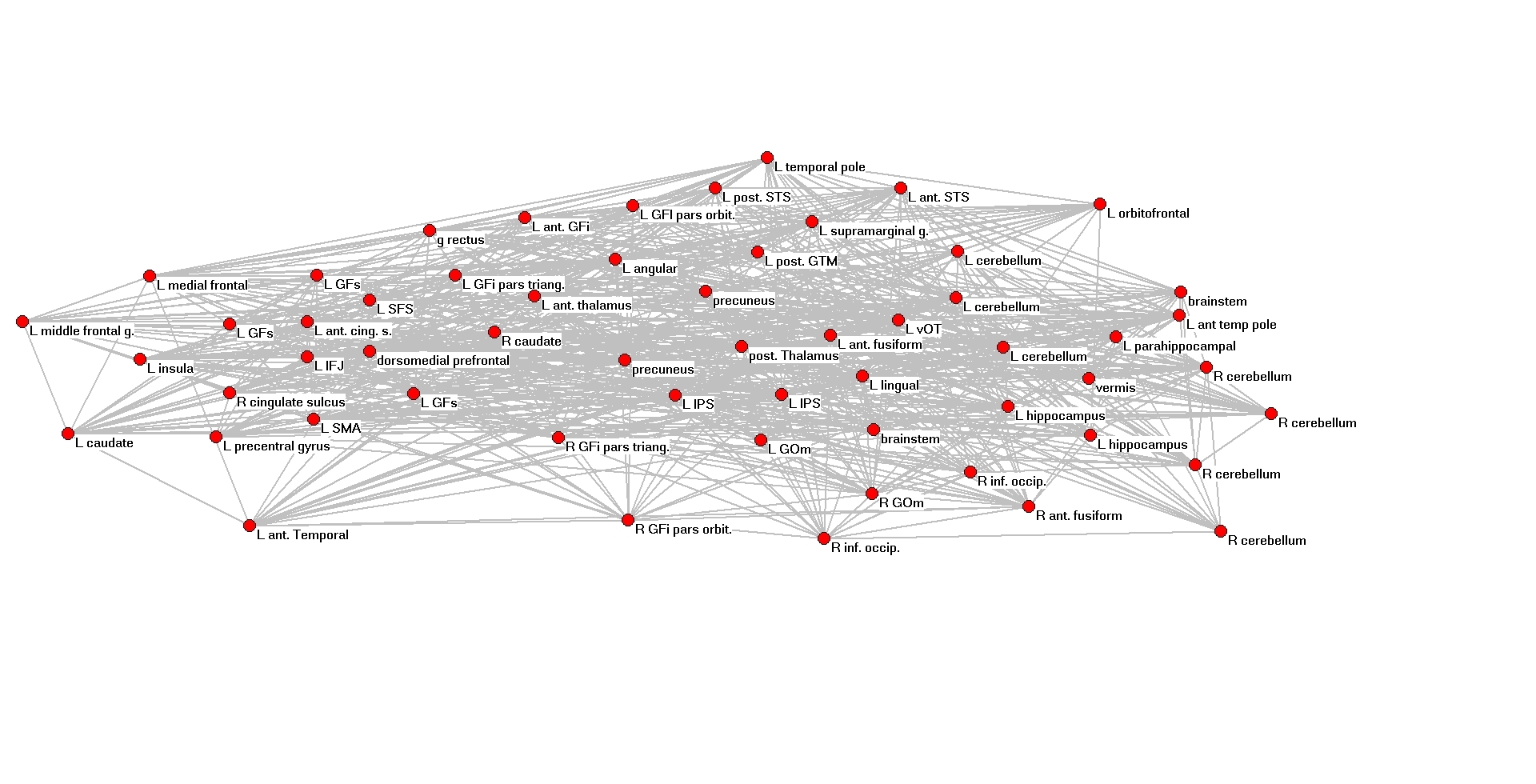

Supplement: S1 Figure — Graph of the associative-semantic network. The connection strength is determined by the partial correlation. Only connections which are significant at uncorrected p0.05 are shown. The density is 42.6%. (JPG) [file pone.0115215.s001.jpg]

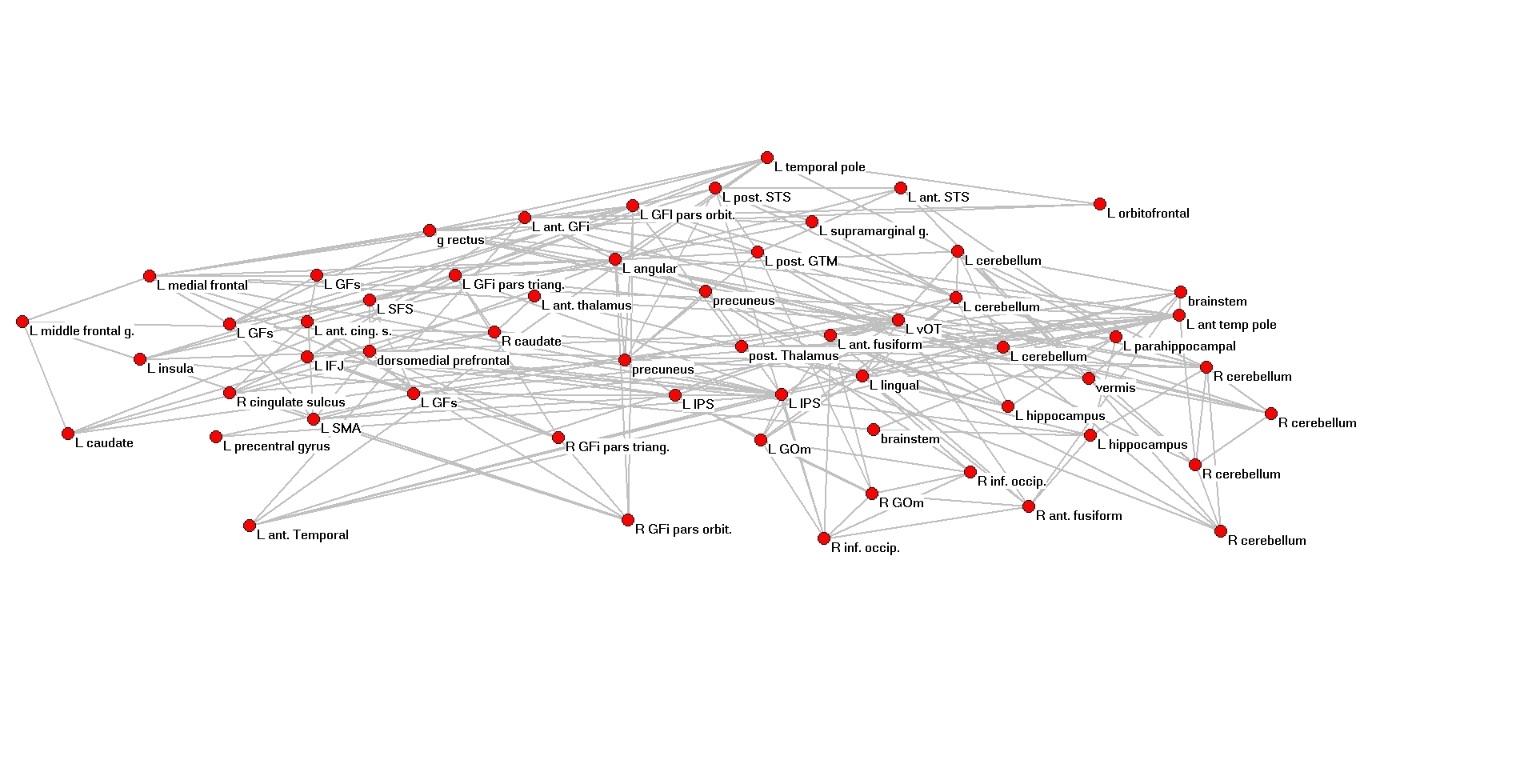

Supplement: S2 Figure — Graph of the associative-semantic network. The connection strength is determined by the partial correlation. Only connections which are significant at corrected (for the number of possible connections) p0.05 are shown. The density is 14%. (JPG) [file pone.0115215.s002.jpg]

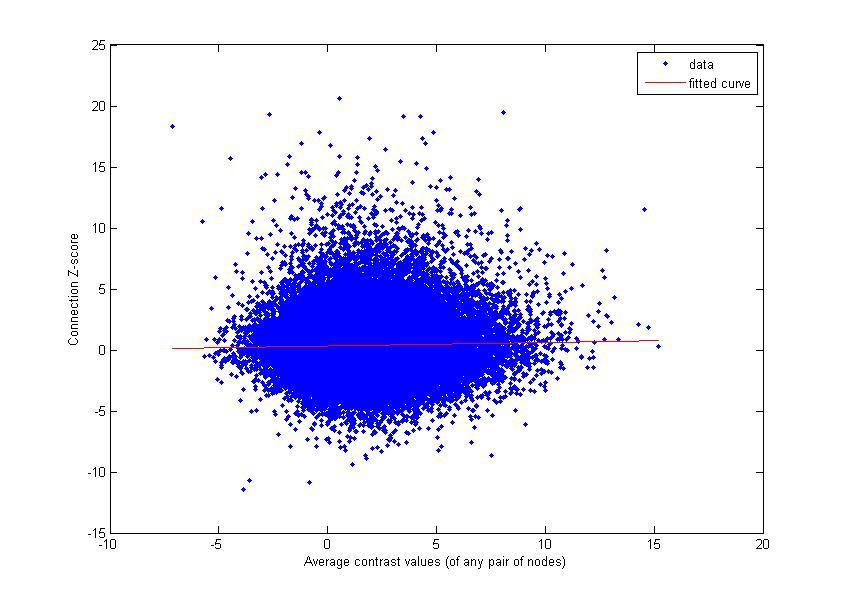

Supplement: S3 Figure — Correlation between the average contrast values (based on the beta values and the main contrast of task) of any pair of nodes and the strength of the functional connectivity (expressed as the Z-values obtained from the partial correlations after a Fisher r-to-z transform) between these nodes to investigate if there is a relation between GLM results and the likelihood of having an edge. Values are plotted for every connection and every subject. The correlation is weak (r = 0.027) but very significant (p). (JPG) [file pone.0115215.s003.jpg]
